# Integrated transcriptome–metabolome analyses reveal regulatory networks underlying soluble solids accumulation in *Capsicum chinense* fruits

**DOI:** 10.1111/tpj.71020

**Published:** 2026-07-09

**Authors:** Wesley Elias Bhering Barrios, Acácio Rodrigues‐Salvador, Rebeca Patrícia Omena‐Garcia, Débora Gonçalves Gouveia, Caris dos Santos Viana, Laise Rosado‐Souza, Pedro Marcus Pereira Vidigal, Diego Mauricio Riaño‐Pachón, Federico Scossa, Alisdair R. Fernie, Wagner L. Araújo, Agustin Zsögön, Adriano Nunes‐Nesi

**Affiliations:** ^1^ National Institute of Science and Technology on Plant Physiology Under Stress Conditions, Departamento de Biologia Vegetal Universidade Federal de Viçosa Viçosa MG 36570‐900 Brazil; ^2^ Max Planck Institute of Molecular Plant Physiology Potsdam‐Golm Germany; ^3^ NuBioMol, Center of Biological Sciences Universidade Federal de Viçosa Viçosa MG 36570‐900 Brazil; ^4^ Laboratório de Biologia Computacional, Evolutiva e de Sistemas, Centro de Energia Nuclear Na Agricultura Universidade de São Paulo Piracicaba Brazil; ^5^ Council for Agricultural Research and Economics (CREA) Research Centre for Genomics and Bioinformatics (CREA‐GB) Via Ardeatina 546 Rome 00178 Italy

**Keywords:** *Capsicum chinense*, non‐climacteric ripening, total soluble solids, multi‐omics integration

## Abstract

The *Capsicum* genus shows remarkable phenotypic diversity, making it an excellent system to study non‐climacteric fruit ripening. Unlike climacteric model species, such as tomato (*Solanum lycopersicum*), the regulatory networks linking transcriptome and metabolome to fruit quality traits remain poorly understood in non‐climacteric crop species. To address this gap, we selected four contrasting *C. chinense* accessions and performed integrated transcriptomic and metabolomic analyses to investigate the regulation of total soluble solids (TSS) accumulation. We profiled 16 922 genes and 63 metabolic features across two fruit developmental stages (immature and mature), including sugars, organic acids, capsaicinoids, and other secondary metabolites. We identified more than 3800 differentially expressed genes and detected strong correlations between gene expression and metabolite levels. Some metabolites, including chlorophylls, carotenoids, and starch, showed consistent temporal trends across genotypes, while others showed genotype‐dependent variation. Our results demonstrate that pepper fruit ripening involves a transcriptional shift toward soluble sugar accumulation, characterized by upregulation of starch‐hydrolyzing enzymes (*CaAMY1/2, CaBAM1*), invertases (*CaINV1, CaCWINV3*), sucrose synthase (*CaSUS2*), and the sugar transporter *CaSWEET10*, alongside downregulation of the starch biosynthetic gene *CaSBE1*. Among these, *CaSUS2*, *CaSWEET10*, and *CaBAM1* emerged as key candidate regulators. These results suggest that coordinated starch degradation and sucrose transport primarily drive TSS increase, while secondary metabolism undergoes independent shifts that characterize other aspects of the ripening process.

## Significance Statement

Non‐climacteric fruit ripening remains less explored than that of climacteric fruits, leaving gaps in our understanding of the regulation of fruit quality in widely consumed crops. Using integrated transcriptome–metabolome analyses of four *Capsicum chinense* accessions, we uncovered a coordinated regulatory network, centered on starch‐hydrolyzing enzymes, invertases, and sugar transporters, which drives total soluble solids accumulation during pepper fruit ripening. These findings reveal candidate genes for marker‐assisted selection and metabolic engineering, supporting quality improvement in non‐climacteric crops and broader agricultural breeding programs.

## INTRODUCTION

The *Capsicum* genus belongs to the Solanaceae family, which includes other major crops, such as tomato (*Solanum lycopersicum*), potato (*S. tuberosum*) and eggplant (*S. melongena*). *Capsicum* species show high phenotypic diversity, particularly for fruit morphology and biochemistry (Jarret et al., 2019; Rosado‐Souza et al., 2015). The five domesticated species *C. annuum* L., *C. chinense* Jacq., *Capsicum frutescens* L., *C. baccatum var. pendulum*, and *C. pubescens* Ruiz and Pavon are widely cultivated for food and industrial products. Whereas tomato serves as a model species to study climacteric fruit ripening (Naves et al., 2019), *Capsicum* fruits are classified as non‐climacteric and follow a different developmental pattern (Giovannoni, 2004; Klie et al., 2014).

Pepper fruits are valued for their health‐promoting compounds and sensory attributes (Scossa et al., 2019). Fruit maturation in *Capsicum* follows distinct phases: fruit set, growth, and ripening, each accompanied by biochemical and physiological changes (Del Giúdice et al., 2023; Hou et al., 2018). Fruit ripening involves coordinated transcriptional and metabolic changes that shape the final chemical composition of the fruit, including the accumulation of sugar and organic acids, alongside processes such as carotenoid biosynthesis, chlorophyll degradation, chromoplast biogenesis, and volatile compound accumulation (Bouvier et al., 1998; Hubbard & Mason Pharr, 1992; Martí et al., 2009). Together, these modifications shape key fruit quality traits, including color, flavor, texture, nutritional value, and total soluble solids (TSS) content (Fayos et al., 2019; Palma et al., 2015; Tartaglia et al., 2021; Tieman et al., 2017).

In higher plants, sink organs accumulate high sugar levels due to the expression of genes associated with sugar transport, accumulation, and metabolism at ripening, like in the pericarp of *C. chinense* fruits, rich in sucrose, fructose, and glucose (Osorio et al., 2012). A concomitant decrease in starch levels is observed during fruit development, as it provides the carbon skeletons for the synthesis of soluble sugars (Hubbard & Mason Pharr, 1992; Osorio et al., 2012). Sucrose synthase (SuSy) and vacuolar acid invertase (AINV) cleave sucrose into UDP‐glucose or hexoses (glucose and fructose), and their activity plays a central role in determining TSS and sugar accumulation in tomato fruits (Beckles et al., 2012; Qin et al., 2016). Consistently, TSS increases during ripening across both climacteric and non‐climacteric fruits (Baxter et al., 2005; Beckles et al., 2012; Martínez et al., 2007; Niklis et al., 2002). Although sugars represent approximately 55% of the TSS content (Helyes et al., 2006), other metabolites such as organic acids (OAs), mostly malate and citrate, dynamically contribute to TSS levels (Batista‐Silva et al., 2018; Etienne et al., 2013) and fruit flavor and quality (Aizat et al., 2014; Flores et al., 2012; Jang et al., 2015; Kader, 2008; Osorio et al., 2012).

During the distinct stages of growth and maturation of pepper fruits, dynamic changes occur in metabolite profiles and gene expression (Giovannoni, 2001; Yang et al., 2024). Notably, malate positively correlates with starch biosynthesis gene expression, suggesting a conserved role in regulating transitory starch metabolism across fruit types (Centeno et al., 2011; Osorio et al., 2012). Although the molecular determinants of TSS during tomato fruit ripening have been explored (Shinozaki et al., 2018; Vallarino et al., 2017), these mechanisms may not be conserved in non‐climacteric fruits like peppers, as the evolutionary lineages leading to tomato and *Capsicum* diverged more than 19 Mya (Särkinen et al., 2013).

In tomato, the acid invertase LIN5 modulates starch levels and TSS content (Fridman et al., 2004); however, the molecular determinants of TSS in *Capsicum* fruits, and their relationship with gene expression during fruit development and ripening remain unclear. Consequently, the extent to which metabolic shifts underlying fruit quality in non‐climacteric species resemble those observed in climacteric fruits such as tomato remains unclear (Carrari & Fernie, 2006; Carrari et al., 2006). An additional factor potentially influencing the metabolic patterns, and thus TSS accumulation on *Capsicum* fruits, is the presence of capsaicinoids: alkaloids responsible for fruit pungency (Naves et al., 2019).

Unlike tomato, where ripening is triggered by ethylene and orchestrated by transcription factors such as *SlRIN*, *SlNOR*, and *SlCNR*, pepper fruits lack a climacteric ethylene burst and appear to rely predominantly on ABA signaling (Hou et al., 2018; Klee & Giovannoni, 2011). Comparative analyses have further shown that only a fraction of ripening‐related genes is shared between the two species (Klie et al., 2014), suggesting that the molecular networks controlling TSS accumulation in tomato cannot be assumed to operate equivalently in *Capsicum*. Although *CaNAC2* has recently been identified as a key transcriptional regulator of pepper ripening (Song et al., 2025), its connections to metabolite dynamics and TSS accumulation remain unclear.

Here, we integrated transcriptomic and metabolomic analyses to assess the determinants underlying TSS accumulation in the pericarp of immature (20 days after anthesis, DAA) and mature (60 DAA) fruits of *C. chinense*. Using two native accessions and two commercial cultivars of *C. chinense*, contrasting in pungency and color, we examined correlations between metabolite profiles and gene expression across 20 and 60 DAA. This approach identified key candidate genes and metabolic pathways potentially driving TSS accumulation in *C. chinense*, providing insights into the molecular basis of non‐climacteric fruit quality.

## RESULTS

### Morphological and metabolic characterization of *C. chinense* fruits at two developmental stages

**Figure 1:**
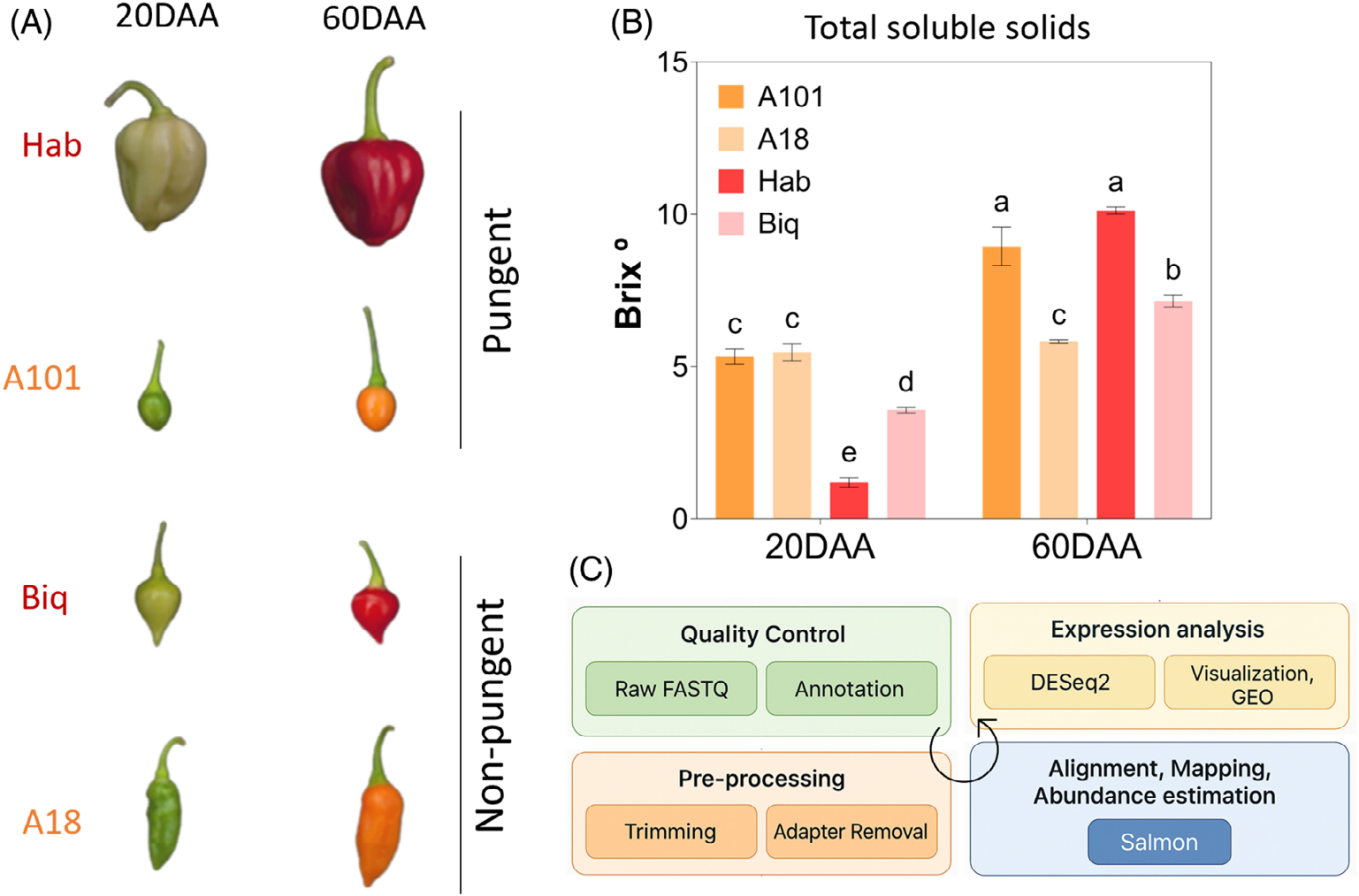
Differential accumulation of total soluble solids during fruit ripening reveals contrasting developmental programs among *C. chinense* accessions. (A) Phenotypic representation of four *C. chinense* accession fruits at two developmental stages (20 and 60 days after anthesis, DAA). Accessions include pungent (Hab and A101) and non‐pungent (Biq and A18) peppers. (B) Total soluble solids (TSS) content in the fruit pericarp at 20 and 60 DAA. Statistical significance was evaluated by two‐way ANOVA followed by Tukey's HSD test (*P* < 0.05). (C) Experimental pipeline. Pericarp samples harvested at 20 and 60 DAA were subjected to RNA‐seq for transcriptome profiling, GC–MS for metabolite analysis, and microplate‐based spectrophotometric enzymatic assays.

**Figure 2:**
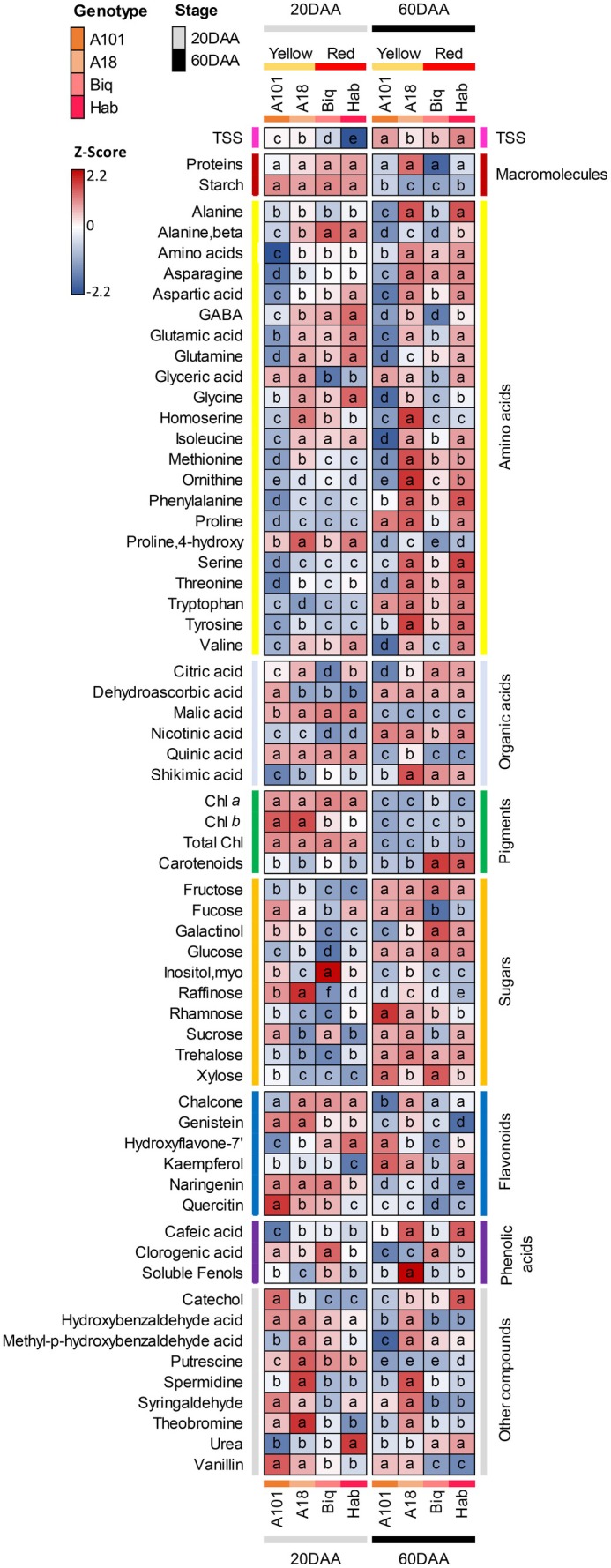
Coordinated accumulation of soluble sugars and genotype‐specific metabolic changes underlies total soluble solids (TSS) gain during *C. chinense* fruit ripening. Heatmap showing the metabolic profile of the pericarp from four accessions (pungent: Hab and A101; non‐pungent: Biq and A18) at 20 and 60 DAA. Colors represent z‐scores (standardized values), indicating how many standard deviations each measurement is above (red) or below (blue) the mean across all groups. Pigments, starch, proteins, amino acids, and soluble phenols were quantified spectrophotometrically; all other metabolites were analyzed via GC–MS. Data were analyzed using two‐way ANOVA followed by Tukey's HSD test (*P* < 0.05). The complete metabolite dataset is available in Tables S1 and S2.

To evaluate phenotypic variability, fruits from four *C. chinense* accessions were sampled at two post‐anthesis stages: 20 and 60 DAA. At full ripeness, the fruits of accessions A18 and A101 showed yellow color and the Hab and Biq exhibited red fruits (Figure 1A). The content of TSS increases through fruit development in all accessions, particularly in red‐fruited ones, except for A18 (Figure 1B). Furthermore, Hab exhibited the lowest TSS content at 20 DAA, followed by a sharp increase at 60 DAA, surpassing the other accessions (Figure 1B). As expected, the carotenoid levels increased during fruit development (60 DAA) in the red‐fruited accessions Hab and Biq, a trend not observed for yellow accessions (Figure 2). Chlorophyll contents decreased across fruit development of all accessions (Figure 2).

Regarding TSS differences among accessions, yellow‐fruited peppers (A18 and A101) showed the highest content at 20 DAA, whereas at 60 DAA the highest values were observed in the pungent Hab and A101 accessions (Figure 1B). As expected, starch content declined markedly from 20 to 60 DAA in all accessions (Figure 2) in contrast to rising TSS levels. Additionally, total free amino acids content increased throughout fruit development, while protein levels remained stable (Figure 2) suggesting enhanced proteolysis or reduced protein synthesis, which may free nitrogen for other processes, but does not directly contribute to TSS.

Metabolic profiling identified 60 metabolites including 46 primary compounds. The relative abundances of these metabolites are summarized in a Z‐score based heatmap (Figure 2). To illustrate stage‐dependent changes, the log2 fold change (LFC) values (60 DAA versus 20 DAA) are represented in Figure S1, while the dynamics of glucose, fructose, sucrose, and starch are presented in Figure S2, Table S1. Among the 21 amino acids analyzed, distinct developmental trends were observed: (i) increasing levels (e.g., alanine, asparagine, methionine, ornithine, phenylalanine, proline, serine, threonine, tyrosine, and tryptophan); (ii) stable levels (e.g., aspartate, glutamate, glycerate, homoserine, isoleucine, valine); and (iii) decreasing levels (e.g., beta‐alanine, GABA, glutamine, glycine, and particularly 4‐hydroxy‐proline). Notably, A101 exhibited the least metabolic variation during ripening, while Hab, Biq, and A18 showed pronounced changes in their profiles.

Six organic acids (OAs) were quantified in the pericarp at two developmental stages (Figure 2). Nicotinate and shikimate increased in all accessions during fruit development. Conversely, citrate and dehydroascorbate increased only in red accessions, with citrate levels decreasing in yellow ones. Malate and quinic acid decreased from 20 to 60 DAA, while shikimate remained constant in all accessions. In general, sugar content increased during development, except for fucose, *myo*‐inositol, and raffinose, which decreased (Figure 2).

Secondary metabolites also varied across developmental stages and accessions. Eighteen metabolites were quantified, including flavonoids (e.g., flavones, chalcones, flavanones, flavonols, hydroxyflavones), phenolic acids, and other phenolic compounds, showing variability between accessions (Figure 2). Overall, flavonoid levels decreased with the onset of ripening, except for kaempferol, which increased. Phenolic acids showed a more heterogeneous pattern: caffeic acid increased in yellow accessions, whereas chlorogenic acid decreased. Other compounds, such as vanillin, and especially putrescine, decreased over time in all accessions. Catechol and theobromine levels also decreased in yellow fruits; however, catechol increased in red ones (Biq and Hab).

### Transcriptome analysis of pericarp tissue in *C. chinense* at two fruit developmental stages

To gain further insight into the molecular mechanisms underlying fruit development, we performed an RNA‐seq analysis on pericarp tissues from four *C. chinense* accessions at two developmental stages: 20 and 60 DAA. A total of 35 842 unique genes were identified, of which 16 922 (Table S2) passed quality filters and were retained for differential gene expression and multivariate analyses.

**Figure 3:**
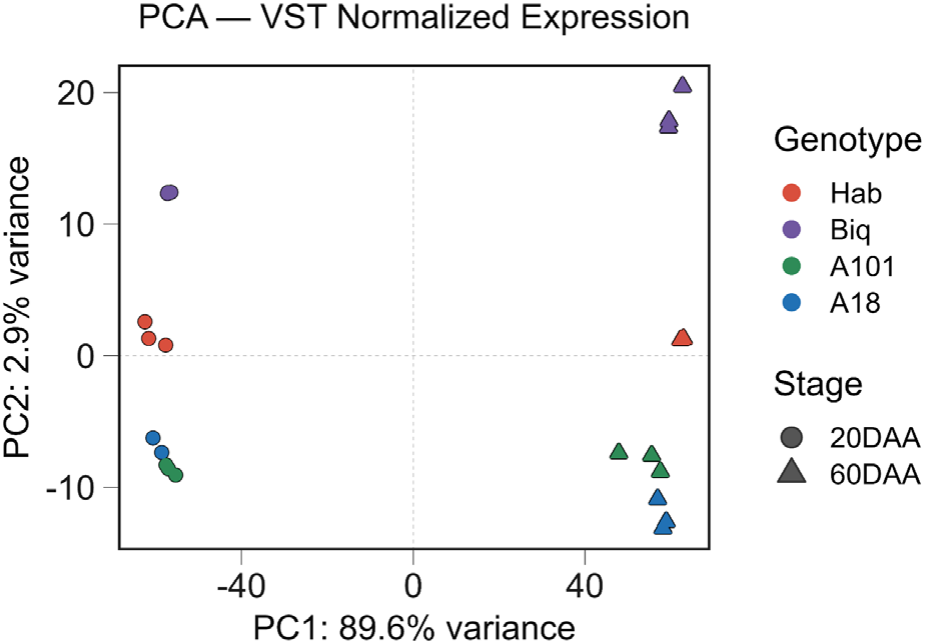
Principal component analysis reveals developmental stage as the primary source of transcriptome variation during *C. chinense* ripening. The PCA plot of 16 922 filtered, VST‐normalized genes from four *C. chinense* accessions (pungent: Hab and A101; non‐pungent: Biq and A18) demonstrates that samples primarily cluster by developmental stage (20 and 60 DAA) along the first dimension. Principal components 1 (PC1) and 2 (PC2) explain 89.6% and 2.9% of the total variance, respectively.

Principal component analysis (PCA) of the expression data revealed that the first two components accounted for 92.5% of the total variance (Figure 3). Replicates clustered consistently, reflecting low intra‐accession variation and confirming the reliability of the dataset. PC1, which explained most of the variance (89.6%), clearly separated samples by developmental stage, with all 20 DAA samples on one side and all 60 DAA samples on the other (Figure 3).

**Figure 4:**
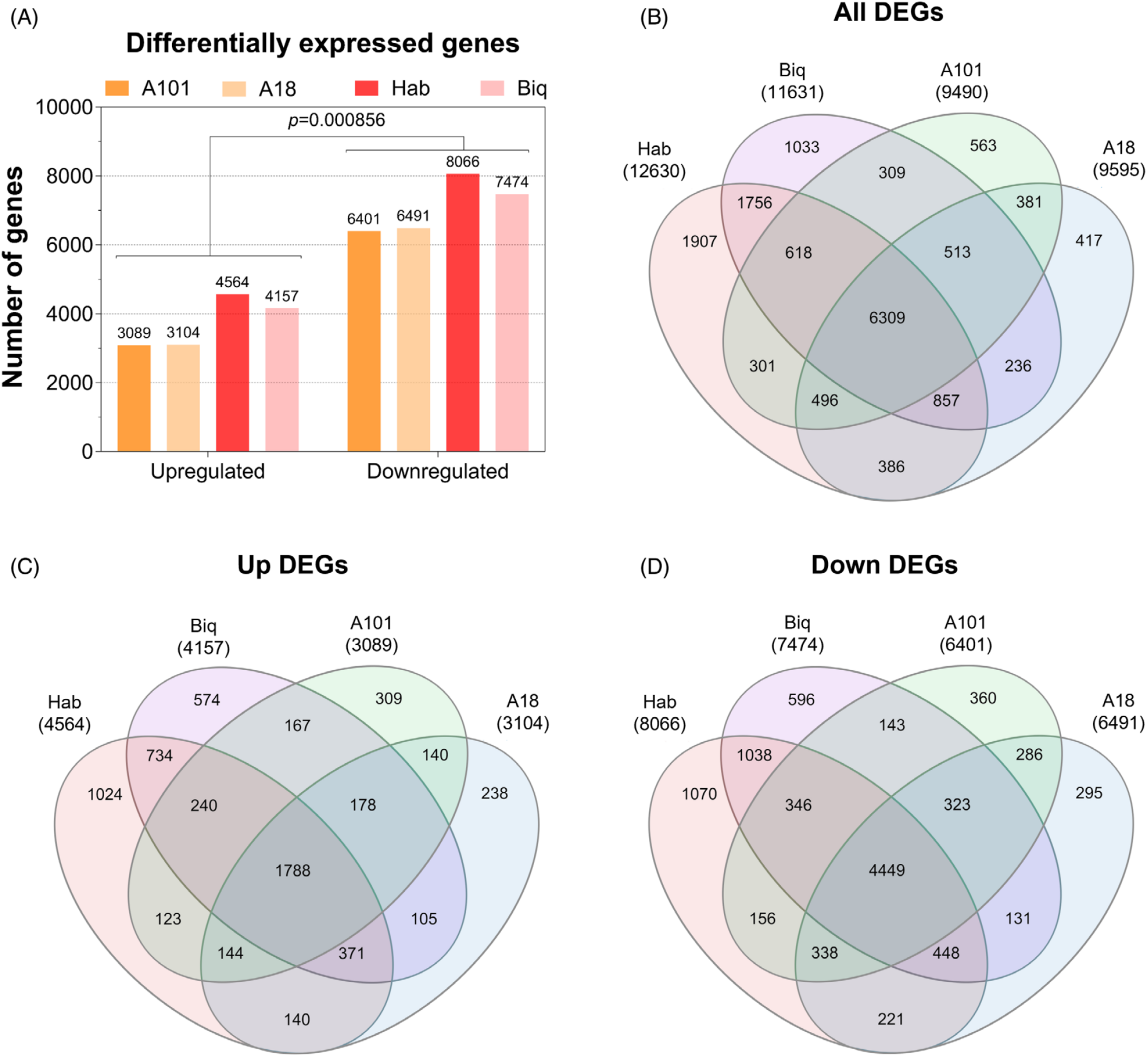
A conserved core of downregulated genes dominates transcriptome remodeling during *C. chinense* fruit ripening. (A) Distribution of differentially expressed genes (DEGs) during fruit ripening across four genotypes (Hab, A101, Biq, and A18). The bar chart compares the total number of upregulated versus downregulated genes within each accession. Statistical significance between the counts of up‐ and downregulated genes was determined using a two‐tailed Welch's *t*‐test, with the resulting *P*‐value displayed above the columns. (B–D) Venn diagrams illustrating the overlap of unique and shared DEGs among the genotypes for (B) All DEGs, (C) Upregulated genes (Up DEGs), and (D) Downregulated genes (Down DEGs). Numbers in parentheses indicate the total DEGs identified per genotype. The core transcriptional ripening program shared across all genotypes encompasses 6309 DEGs (1788 upregulated and 4449 downregulated). Notably, Habanero exhibited the most extensive, genotype‐specific transcriptional shifts (12 630 total DEGs), followed by Biquinho (11 631), A18 (9595), and A101 (9490).

Differential expression analysis across the developmental stages for each accession revealed distinct expression dynamics. The number of significantly (FDR <0.05) downregulated genes from 20 to 60 DAA was 8066 for Hab, 6401 for A101, 7474 for Biq, and 6491 for A18. The corresponding count of upregulated genes was 4564, 3089, 4157, and 3104 for Hab, A101, Biq, and A18, respectively (Figure 4A). Overall, red‐fruited accessions (Hab and Biq) showed a greater number of both down‐ and upregulated genes compared with yellow‐fruited accessions (A101 and A18).

Because mapping against the available *Capsicum chinense* reference genome (PI159236) yielded low alignment rates, we also aligned the RNA‐seq reads to the *Capsicum annuum* reference (Zunla‐1). This resulted in a substantial improvement, from an average mapping rate of 53.82 to 81.27%, indicating better read assignment and reference compatibility (Figure S3). From the cross‐mapping analysis, Spearman correlation coefficients between PI159236 and Zunla‐1 were consistently high (ρ > 0.97 for all conditions), suggesting that the overall expression patterns are strongly conserved despite the cross‐species mapping (Figures S4 and S5). When the analysis was restricted to genes with moderate expression levels (25th–75th percentiles of PI159236 TPM), correlations were consistently high (ρ > 0.84 for all conditions), supporting that these associations are not driven by outliers. Genes identified as differentially expressed (DEGs) in Zunla‐1 between developmental stages (20 versus 60 DAA) were highlighted in the scatter plots. These DEGs neither formed a distinct cluster nor systematically deviated from the 1:1 trend, and their correlation coefficients were also high (ρ typically >0.88), indicating that genes undergoing regulation in one accession still maintain coordinated expression with the others.

The small proportion of outlier DEGs from the cross‐mapping likely reflects natural variation in sequence conservation between *C. chinense* and *C. annuum*, supporting the absence of systematic mapping bias in our cross‐species approach. For the small subset of genes with divergent quantifications, however, it remains unclear whether these differences arise from cross‐species mapping limitations or from sequence‐level issues in the flagged *C. chinense* genome assembly (contaminated). Venn diagrams further illustrate the patterns of gene expression regulation across genotypes. A total of 4449 downregulated and 1788 upregulated genes were shared by all accessions (Figure 4B–D), suggesting that a substantial proportion of gene regulation during pericarp development is governed by global downregulation. Red‐fruited accessions shared a greater number of DEGs (6281 down and 3133 upregulated genes) than yellow‐fruited ones (5346 upregulated and 2250 downregulated genes, Figure 4B–D).

In addition, red‐fruited accessions exhibited the highest number of unique DEGs and a higher increase in TSS from 20 to 60 DAA, with Hab and Biq showing 1907 and 1033 exclusive DEGs, respectively. In contrast, A101 and A18 exhibited 563 and 417 unique DEGs, respectively (Figure 4C). Red‐colored Hab and Biq accessions shared 9540 genes, while yellow‐colored ones shared 7699. In contrast, 7724 genes were shared between pungent accessions (Hab and A101) and 7915 between the non‐pungent ones (Biq and A18).

### Identification of key genes associated with total soluble solids content in pepper fruits

**Figure 5:**
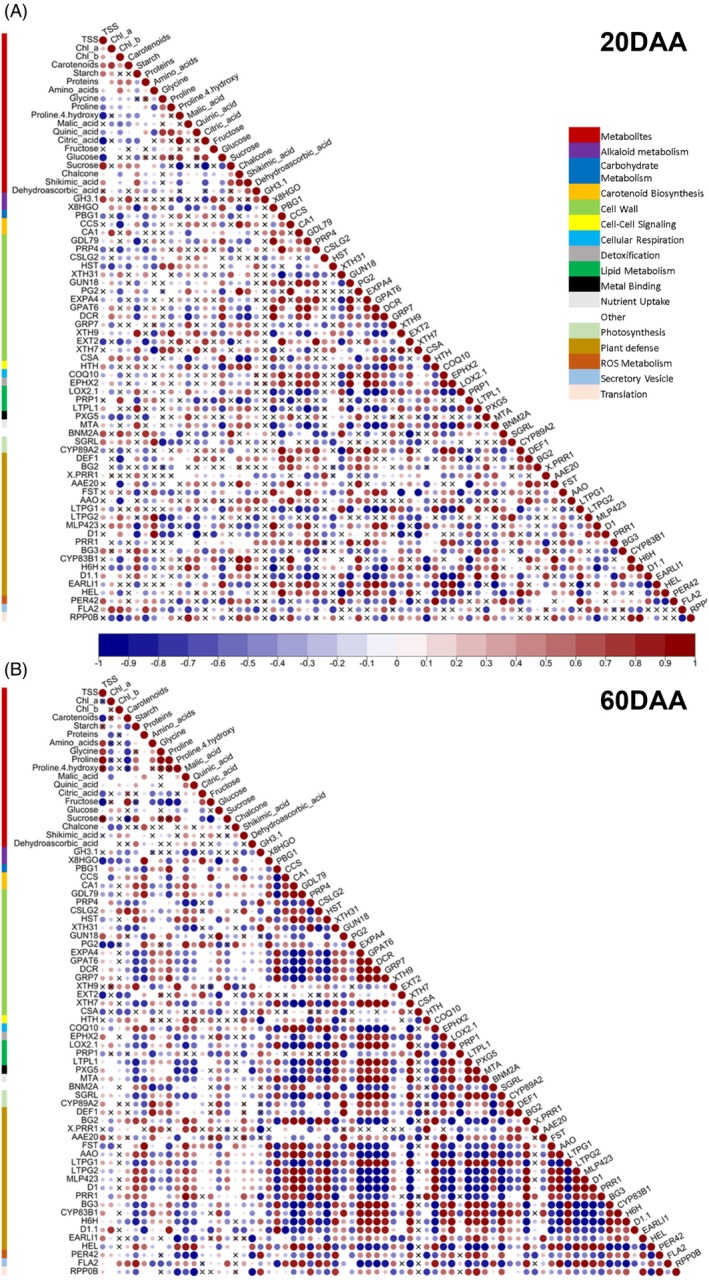
Stage‐dependent reorganization of the total soluble solids (TSS) correlation network reveals coordinated metabolic and transcriptional shifts at 60 DAA. Correlogram of Pearson correlation coefficients (PCCs) between selected genes and metabolite profiles from four *C. chinense* accessions at (A) 20 DAA and (B) 60 DAA. Non‐significant correlations (FDR ≥0.05) are marked with an “X,” whereas all colored cells represent significant correlations (FDR <0.05). Color intensity and sphere size indicate the magnitude of the correlation (larger spheres indicate stronger correlations). Metabolites and gene groups are color‐coded by their respective functional categories.

To explore the molecular basis of TSS accumulation in *Capsicum* fruits, we performed Pearson correlation analyses between the transcriptomic and metabolomic profiles at 20 and 60 DAA to identify genes potentially associated with TSS (Figure 5, Tables S3 and S4 for raw data). The strongest correlations occurred at 60 DAA; however, some significant correlations were also detected at 20 DAA. This trend was consistent for correlations between TSS and both metabolites and gene expression.

At 20 DAA, TSS correlates positively with carotenoids, starch, sucrose, shikimate, and several genes, including *GH3.1* (indole‐3‐acetic acid amido synthetase), *HTH* (protein HOTHEAD‐like), *BNM2A* (BURP domain containing protein BNM2A‐like), *SGRL* (protein STAY GREEN chloroplastic‐like), *BG3* (glucan‐endo‐1,3‐β‐glucosidase 3), and *PER42* (peroxidase 42). In contrast, negative correlations were found between TSS and glycine, proline, 4‐hydroxyproline, citrate, glucose, *PBG1* (β‐galactosidase‐like), *PRP4* (proline‐rich protein 4‐like), *HST* (shikimate O hydroxycinnamoyl transferase‐like), and *EXT2* (extensin 2‐like) (Figure 5A).

At 60 DAA, TSS correlates positively with total free amino acids, glycine, proline, *GUN18* (endoglucanase 18‐like), *EPHX2* (bifunctional epoxide hydrolase 2‐like), *AAE20* (benzoate CoA ligase peroxisomal‐like), and *EARLI1* (lipid transfer protein EARLI 1‐like). Negative correlations were found with carotenoids, citrate, fructose, and genes including *X8HGO* (X8‐hydroxygeraniol dehydrogenase‐like), *PG2* (polygalacturonase 2), *EXT2*, *CSA* (cellulose synthase A catalytic subunit 2 UDP‐forming‐like), *EPHX2* (bifunctional epoxide hydrolase 2‐like), and *FST* (flower‐specific defensin‐like, Figure 5B).

**Figure 6:**
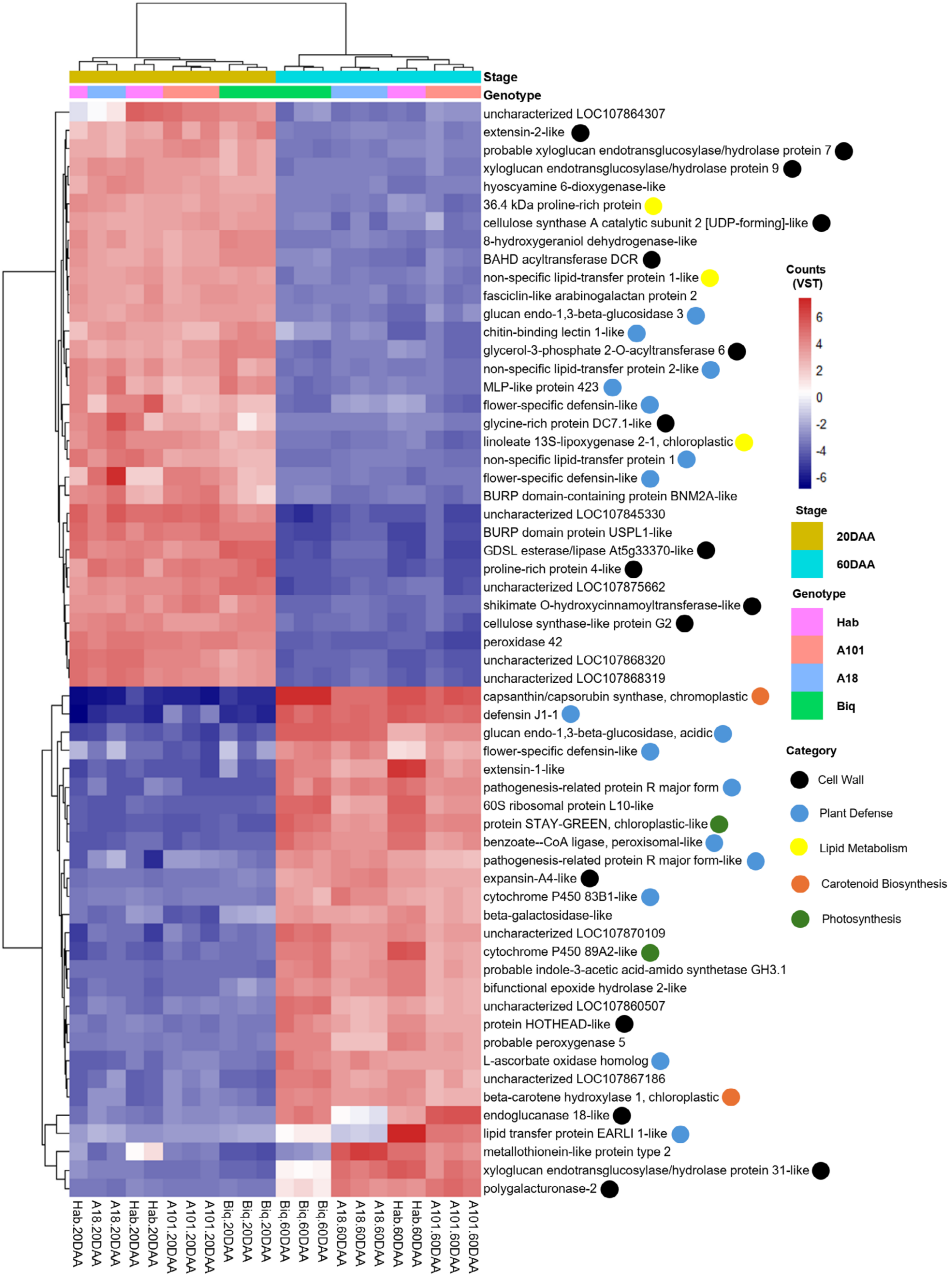
Transcriptional reprogramming drives the transition from fruit expansion to ripening through coordinated shifts in sugar metabolism and cell wall remodeling in *C. chinense*. Heatmap of VST‐normalized expression values for the 60 most variable genes across four *C. chinense* accessions (Hab, A101, A18, and Biq) at 20 and 60 DAA. Samples and genes were hierarchically clustered using Euclidean distance with complete linkage. The color scale represents expression levels, ranging from low (blue, −6) to high (red, +6). Two major clusters are evident: an upper cluster, highly expressed at 20 DAA, enriched in cell wall biosynthesis, lipid transfer, and structural genes; and a lower cluster, induced at 60 DAA, encompassing cell wall loosening enzymes, chromoplast differentiation factors, and hormone metabolism genes (including auxin inactivation). Specific gene annotations are indicated on the right axis. Colored circles adjacent to each gene name indicate its functional category: black, cell wall; blue, plant defense; yellow, lipid metabolism; orange, carotenoid metabolism; and green, photosynthesis. The category terminology is consistent with the curated annotation framework used for this gene set.

To further identify genes with dynamic expressions during fruit development, we constructed a heatmap of the top 60 most variably expressed genes across samples (Figure 6, Table S5). Among the 60 genes, 51 were functionally annotated, representing 16 genes involved in plant defense, 16 in cell wall metabolism, three in lipid metabolism, two in carotenoid biosynthesis, two in photosynthesis, two in alkaloid metabolism, and the remaining 10 distributed across carbohydrate metabolism, cellular respiration, detoxification, metal binding, nutrient uptake, ROS metabolism, secretory vesicle trafficking, translation, and cell–cell signaling (Table S5).

**Figure 7:**
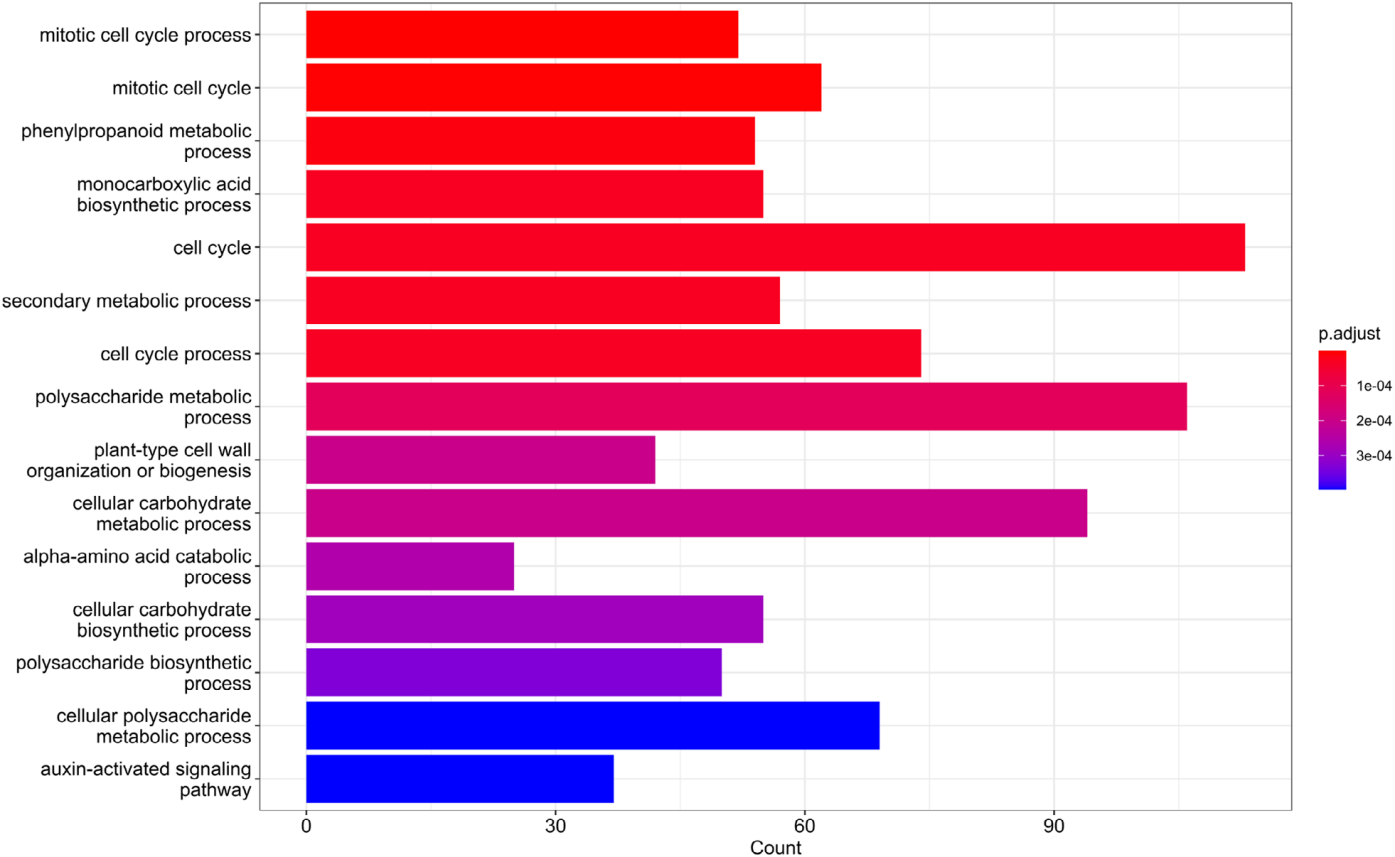
Gene ontology enrichment of DEGs highlights the activation of carbohydrate metabolism and cell wall remodeling during ripening. Horizontal bar chart showing significantly enriched biological processes (GO terms) among the core set of shared genes differentially expressed between 20 and 60 DAA across all four *C. chinense* accessions. Bars represent the total number of genes (count) associated with each biological process. Color intensity indicates statistical significance based on Benjamini–Hochberg adjusted *P*‐values. The top 15 most significantly enriched processes are displayed, reflecting the coordinated suppression of early developmental traits and the activation of ripening‐associated pathways underlying TSS accumulation.

These genes together with all common DEGs across genotypes were functionally annotated to determine their involvement in metabolic processes relevant to fruit pericarp development (Figure 7). The analysis revealed upregulation of genes involved in sugar metabolism and cell wall remodeling during ripening, with several genes showing differential expression at both time points, suggesting potential genetic mechanisms influencing fruit ripening dynamics in these *C. chinense* accessions.

The identified genes spanned a range of biological functions, including transcriptional regulation, signal transduction, and various metabolic pathways essential to the regulation of fruit ripening. Several genes related to hormone biosynthesis, sugar transport, and cell wall metabolism were differentially expressed. Among these, soluble sugar accumulation genes were highlighted (Table 1). Genes encoding starch‐degrading enzymes (*CaAMY1/2, CaBAM1*) and sucrose‐cleaving invertases (*CaINV1, CaCWINV3*) were strongly upregulated, alongside the sucrose synthase *CaSUS2* and the sugar transporter *CaSWEET10*, collectively promoting carbohydrate mobilization and sugar loading into fruit tissues. Conversely, downregulation of *CaSBE1* and *CaSUS1* further supports the reorientation of carbon metabolism away from starch biosynthesis. Collectively, these findings highlight the complexity of sugar accumulation during pepper fruit ripening, involving coordinated expression of both known and novel genes.

**Table 1:** Differential expression and functional annotation of key genes associated with carbohydrate metabolism related to total soluble solids (TSS) accumulation during ripening in *Capsicum chinense* fruit pericarp

Gene symbol	Name	Gene ID	Metabolic or regulatory role	A18 LFC	FDR	A101 LFC	FDR	Biq LFC	FDR	Hab LFC	FDR
*CaNAC2*	NAC domain‐containing protein 2	LOC107867776	Ripening‐associated transcription factor that acts as a positive regulator of fruit ripening	5.36	1.5E‐160	4.67	6.9E‐157	6.40	7.2E‐283	6.85	8.8E‐254
*CaSWEET10*	Sugars Will Eventually be Exported Transporter 10	LOC107856010	Mediates bidirectional sugar transport, particularly sucrose efflux between cells	6.27	4.6E‐20	7.56	7.1E‐36	8.04	5.4E‐39	10.45	3.5E‐48
*CaAMY1*	Alpha‐amylase 1	LOC107867836	Degrades stored starch, releasing soluble sugars to support energy supply and fruit ripening	2.17	7.6E‐11	1.98	1.9E‐10	3.37	4.2E‐27	4.49	2.8E‐39
*CaAMY2*	Probable alpha‐amylase 2	LOC107866823	Hydrolyzes starch by cleaving α‐1,4 glycosidic bonds, contributing to carbohydrate mobilization	1.69	3.8E‐22	2.04	1.8E‐39	2.31	4.5E‐49	2.21	3.0E‐36
*CaBAM1*	Beta‐amylase 1	LOC107864541	Degrades starch from non‐reducing ends, releasing sugars during fruit ripening	2.77	1.6E‐07	2.58	5.2E‐08	3.21	1.6E‐11	3.36	2.2E‐10
*CaCWIN3*	Cell wall invertase 3	LOC107850632	Hydrolyzes sucrose in the apoplast, producing glucose and fructose for cellular growth and metabolism	1.99	3.6E‐50	1.49	3.1E‐36	2.43	8.0E‐92	2.20	2.4E‐60
*CaINV1*	Acid beta‐fructofuranosidase AIV‐18	LOC107863437	Irreversibly converts sucrose into glucose and fructose in the vacuole, regulating sugar accumulation	4.69	1.3E‐58	2.96	1.5E‐30	5.15	4.8E‐89	4.74	6.6E‐61
*CaSBE1*	1,4‐alpha‐glucan‐branching enzyme‐like	LOC107866797	Forms α‐1,6 linkages in amylopectin during starch biosynthesis	−7.06	0.0E+00	−6.34	0.0E+00	−7.08	0.0E+00	−6.36	4.9E‐263
*CaSUS1*	Sucrose synthase 1	LOC107840883	Catalyzes the reversible cleavage of sucrose, providing substrates for metabolism and biosynthetic processes	−3.39	7.0E‐64	−2.31	1.5E‐38	−2.93	5.6E‐61	−2.96	6.6E‐50
*CaSUS2*	Sucrose synthase 2	LOC107864054	Participates in sucrose metabolism through its reversible conversion into fructose and UDP‐glucose	3.93	1.7E‐130	3.18	9.3E‐108	4.20	6.4E‐184	4.49	8.1E‐170
*CaSuSy*	Sucrose synthase‐like	LOC107877893	Sucrose synthase isoform involved in sucrose degradation and utilization	−9.56	3.3E‐70	−11.22	1.1E‐26	−10.99	9.36E‐36	−9.80	3.59E‐12

The table lists the selected genes, including gene symbol, name, Gene ID, and a curated description of their metabolic or regulatory role. Differential expression between 60 DAA and 20 DAA was estimated with DESeq2, and log2 fold change (LFC) and adjusted *P*‐value (FDR) are shown for each genotype. Colors represent differential expression magnitude, with red being upregulated and blue as downregulated genes.

### Overrepresentation analysis of shared significant DEGs in pepper fruits

To investigate the biological processes associated with fruit ripening in the four *C. chinense* accessions, we performed an overrepresentation analysis using the dataset comprising 6309 shared significant DEGs (Figure 7). This dataset includes 4449 downregulated and 1788 upregulated genes across accessions during the transition from 20 to 60 DAA (Figure 4).

The top 15 significantly enriched biological pathways based on LFC (60/20 DAA) were highlighted, reflecting key processes regulated during pepper development. The pathways include mitotic cell cycle process, mitotic cell cycle, phenylpropanoid metabolic process, monocarboxylic acid biosynthetic process, cell cycle, cell cycle process, secondary metabolic process, polysaccharide metabolic and biosynthetic process, plant‐type cell wall organization or biogenesis, cellular carbohydrate metabolic and biosynthetic process, amino acid catabolic process, cellular polysaccharide metabolic process, and auxin‐activated signaling pathway. Among these, genes involved in cell cycle regulation, primary metabolism (including carbohydrate and polysaccharide metabolism), secondary metabolism, cell wall remodeling, and auxin signaling were the most significantly overrepresented across all accessions (Figure 7).

These results underscore the coordinated regulation of developmental and metabolic pathways during *C. chinense* fruit ripening. The enrichment of carbohydrate metabolism and polysaccharide remodeling terms provides pathway‐level support for the TSS accumulation observed at 60 DAA. Notably, the overrepresentation of auxin‐activated signaling among downregulated genes is consistent with a decline in auxin activity during the transition to fruit maturity. This observation raises the hypothesis that reduced auxin signaling may release transcriptional repression of sugar metabolism and cell wall loosening programs, thereby contributing to TSS accumulation, which remains to be functionally validated.

## DISCUSSION

### Metabolic and transcriptomic dynamics during fruit development

Comparative analyses of pepper fruits between 20 and 60 DAA revealed distinct transcriptional and metabolic profiles. Although transcription and metabolism are not directly coupled (Caldana et al., 2023), their coordinated changes highlight a strong impact of developmental stage on the regulation of fruit ripening (Seymour et al., 2013; Villa‐Rivera & Ochoa‐Alejo, 2021). Fruit developmental stage had a greater impact on transcriptional and metabolic profiles than genotype, and the grouping of DEGs based on fruit color appeared more pronounced than grouping by pungency, suggesting that fruit color is more strongly correlated with gene expression than pungency; however, this inference is based on DEG counts and should be interpreted with caution, especially considering the potential bias caused by the use of the red‐fruited *C. annuum* as a reference genotype.

Organic acids shape fruit flavor and vary dynamically in tomato fruit development (Araújo et al., 2011; Carrari & Fernie, 2006; Centeno et al., 2011). However, unlike tomato, in which a decline in malate and fumarate is associated with ripening (Morgan et al., 2012), *C. chinense* exhibited lower fumarate levels and a decrease in malate (Figure S1). Furthermore, in contrast with tomato (Osorio et al., 2012), no clear transcriptional correlation was found between malate levels and starch‐related genes, pointing toward post‐transcriptional regulation in *C. chinense*. Interestingly, the ripening‐associated increase in free amino acid levels, despite stable total protein content (Figure 2), suggests enhanced protein turnover and/or reduced protein synthesis, reflecting nitrogen remobilization for maturation‐related processes (Gomez‐Zepeda et al., 2026; Ling et al., 2021). These metabolic shifts likely drive carbon–nitrogen balance adjustments rather than directly contributing to TSS accumulation.

Photosynthesis‐related pathways and starch biosynthesis are highly active at early fruit developmental stages and decline as ripening progresses, concomitantly with chlorophyll degradation and carotenoid accumulation (Osorio et al., 2012; Sadali et al., 2019), a pattern consistently observed in the present study (Figure 2; Table S5). This metabolic shift redirects carbon from storage pools toward soluble sugar accumulation, with glucose, fructose, and maltose contents increasing as starch decreases (Carrari & Fernie, 2006; Centeno et al., 2011). At 60 DAA, the downregulation of sucrose degradation genes (i.e., SuSy, invertases), alongside upregulation of α‐ and β‐amylases, suggests that coordinated starch breakdown and sugar import are the primary drivers of sugar accumulation in mature *C. chinense* fruits (Beckles et al., 2012).

TSS content is determined not only by soluble sugars but also by OAs and starch‐derived metabolites (Centeno et al., 2011; Mounet et al., 2009; Tiessen et al., 2002). In tomato, sugars account for around 55% of TSS, with starch breakdown and phloem‐derived sucrose contributing to TSS rise during ripening (Aizat et al., 2014; Beckles et al., 2012; Helyes et al., 2006; Martínez et al., 2007). Consistently, the positive correlation between *CaSUS2* expression and TSS, alongside the inverse relationship between starch and TSS (Figure 2), supports sugar accumulation as a coordinated outcome of sucrose mobilization and starch breakdown in ripening fruits (Durán‐Soria et al., 2020; Fernie et al., 2006). Beyond these canonical pathways, contrasting correlations between cell wall remodeling genes with TSS suggest a prominent role for apoplastic carbohydrate hydrolysis at early ripening stages (Figure 5, Shen et al., 2018), while negative correlations between hexokinase expression and TSS at 60 DAA may reflect reduced sugar sensing as ripening progresses (Halford et al., 1999). Together, these findings suggest that TSS accumulation in *C. chinense* results from the integrated action of sucrose metabolism, starch remobilization, and sugar signaling. However, the exact contribution of alternative metabolites beyond carbohydrates and OAs remains to be elucidated.

### Overrepresented pathways in pepper fruit ripening

Overrepresentation analysis of shared differentially expressed genes (DEGs, Figure 7) highlights that TSS accumulation is tightly integrated with broader developmental shifts rather than being an isolated metabolic event. While primary metabolism directly drives TSS through sucrose cleavage and starch degradation, our data suggest structural remodeling also plays a critical role. Enriched pathways related to cell wall organization and polysaccharide metabolism imply that changes in fruit texture and firmness likely facilitate sugar transport and compartmentalization (Schaffer et al., 2007; Shi et al., 2023). This metabolic sink is further coordinated by hormonal cues, particularly auxin‐activated signaling, which modulates sugar metabolism during fruit ripening (Forlani et al., 2019).

Conversely, prominent shifts in secondary metabolism do not appear to directly impact TSS. The phenylpropanoid pathway, which fuels the production of flavonoids and capsaicinoids, represents a specialized carbon sink (Fayos et al., 2019; Mazourek et al., 2009; Ninkuu et al., 2025; Schaart et al., 2013). Capsaicinoid synthesis, catalyzed by *CaPun1* and absent in non‐pungent varieties, likely influences the overall metabolic balance rather than TSS itself (Jiang et al., 2025; Naves et al., 2022; Ogawa et al., 2015). Similarly, organic acid dynamics, while impacting overall flavor (Liu et al., 2019), operate alongside sugar accumulation. Together, these findings suggest that TSS in *C. chinense* is a complex trait governed by the coordinated interplay of cell wall remodeling, primary metabolism, and hormonal control, while functioning independently of pungency‐related secondary sinks.

Intriguingly, contrasting hormonal architectures of ripening may underlie the divergent transcriptional regulation of sugar metabolism genes observed between tomato and pepper. In tomato, autocatalytic ethylene signaling promotes a rapid and coordinated ripening program that drives sharp changes in soluble sugar mobilization and organic acid degradation (Hu et al., 2024; Yang et al., 2021). By contrast, the limited induction of ethylene biosynthesis genes in pepper shifts the regulation of sugar and organic acid metabolism toward ABA‐dependent pathways (Bai et al., 2021; Mou et al., 2016; Osorio et al., 2012; Wang, Wen, et al., 2025). As ABA accumulates progressively, it regulates responsive transcription factors such as *AREB/ABF* that control key sugar metabolism enzymes (Du et al., 2024; Ma et al., 2017; Zhu et al., 2023), resulting in more gradual metabolic adjustments than those observed in tomato. Together, reduced ethylene signaling combined with the predominant ABA influence likely shapes the distinct metabolic landscape governing fruit flavor and quality development in pepper.

### Temporal reprogramming of gene expression and metabolite levels

At 20 DAA, positive correlations between TSS and pigments, starch, and sucrose (Figure 5A) reflect a developmental stage in which active photosynthesis sustains carbon supply and builds reserves (Klie et al., 2014; Wang, Li, et al., 2025). Negative correlations with amino acids (glycine and proline, 4‐hydroxyproline) and TCA cycle intermediates (quinic acid and citric acid) are consistent with the rise in OAs and nitrogen‐containing compounds, reflecting carbon allocation toward anabolic processes rather than soluble sugar pools (Zhang et al., 2020).

At the transcriptional level, positive correlations with cell wall‐modifying genes (*CaXTH31*, *CaXTH9*) and lipid transfer proteins (*CaLTPG1*, *CaLTPL1*) suggest that early fruit expansion and surface remodeling are coordinated with sugar import (Figure 5A, Cosgrove, 2005; García‐Coronado et al., 2022; Li et al., 2023). Conversely, negative associations with structural genes (*CaPRP4*, *CaCSLG2*, *CaEXT2*) and *CaHST* suggest selective carbon diversion away from structural deposition (Lamport et al., 2011). A notable finding is that *CaGH3.1* shows a strong positive correlation with TSS at 20 DAA (Figure 5A), supported by its extremely low transcript levels at this stage (Table S5). GH3 enzymes conjugate free IAA to amino acids, lowering active auxin levels, and GH3‐mediated auxin inactivation has been identified as a prerequisite for ripening onset and progression in several fleshy fruits (Bernales et al., 2019; Böttcher et al., 2011; Peat et al., 2012; Sravankumar et al., 2018). The sharp increase in *CaGH3.1* expression at 60 DAA (Table S5), therefore, suggests that auxin attenuation via this gene may act as a staged hormonal switch that first sensitizes, and later consolidates, a ripening‐permissive state favoring sugar accumulation in *C. chinense*. Further studies will be required to test this mechanism directly.

By 60 DAA, the shift from negative to positive correlations between TSS and total free amino acids (Figure 5B) is consistent with amino acid catabolism feeding the TCA cycle and carbohydrate metabolism via the GABA shunt. This pathway connects nitrogen metabolism to sugar pools as photosynthate import declines (Fait et al., 2008). Concurrently, the persistent positive correlations with *CaEXPA4*, *CaXTH7, and CaPXG5* suggests that cell wall remodeling activity is redirected toward apoplastic sugar loading rather than cell expansion (Cosgrove, 2005). Furthermore, strong positive correlations with defense and cuticle biosynthesis genes (*CaEPHX2*, *CaCYP89A2*, *CaGPAT6*, *CaDCR*) support the hypothesis of intensified barrier formation and oxidative stress responses accompanying late‐stage sugar accumulation (Figure 5B) (Li et al., 2022). Conversely, the negative correlations observed with *CaEXT2*, *CaPG2*, and carotenoids suggest a metabolic transition away from structural biosynthesis in favor of solute loading (Durán‐Soria et al., 2020; Xue et al., 2020). Intriguingly, this uncoupling suggests that TSS accumulation is regulated independently of carotenoid build‐up during late ripening stages.

Overall, TSS accumulation in *C. chinense* follows a temporal stage logic: at 20 DAA it is primed by photosynthetic carbon supply and cell wall loosening, while at 60 DAA, it is supported by starch and amino acid catabolism together with continued cell wall remodeling. This coordinated metabolic and transcriptional reprogramming, operating without an ethylene burst of climacteric species, highlights possible auxin homeostasis and nitrogen–carbon crosstalk as underexplored regulatory nodes in non‐climacteric fruit quality (Fuentes et al., 2019; Giovannoni, 2001; Klee & Giovannoni, 2011; Kou et al., 2021).

### Transcriptional reprogramming during maturation

In *C. chinense* pericarp, the 60 most variably expressed genes cluster into two groups: one, highly expressed at 20 DAA and downregulated by 60 DAA, and another with low early expression that rises sharply in ripe fruit (Figure 6; Table S5). These complementary trajectories reflect a coordinated shift from cell wall fortification and defense toward metabolic remobilization and secondary metabolite biosynthesis during fruit maturation, paralleling TSS accumulation.

The downregulated gene set includes extensin‐2‐like structural glycoproteins, xyloglucan endotransglucosylase/hydrolases, β‐1,3‐glucanases and proline‐rich proteins, all implicated in reinforcing cell wall integrity and mediating defense responses (Brummell, 2006; Cosgrove, 2005; Zuo et al., 2019). Their downregulation at 60 DAA coincides with cell wall loosening and fruit softening, processes associated with enhanced solute accumulation during ripening (Berry et al., 2019; Brummell, 2006). Concomitant repression of chitin‐binding lectins and defensin‐like proteins may suggest a late‐stage reduction in defense investment and possible reallocation of nitrogen and carbon from protective proteins toward primary metabolism, although the mechanistic link remains to be established (Desnoues et al., 2014).

Conversely, upregulated genes during maturation include capsanthin/capsorubin synthase, lipoxygenases, GDSL acyltransferases and probable peroxygenases, consistent with enhanced carotenoid and oxylipin biosynthesis underlying fruit pigmentation and flavor (Figure 6, Gonzalez‐Gordo et al., 2022; Song et al., 2022). The induction of β‐galactosidase‐like and β‐carotene hydroxylase enzymes may contribute to cellular osmotic balance, favoring solute accumulation and maintenance of high TSS levels (Jin et al., 2006; Liu et al., 2019; Villa‐Rivera et al., 2022). Activation of glucanases and GDSL lipases may also facilitate redistribution of carbon and solutes within fruit tissues, supporting the accumulation of soluble compounds associated with higher TSS (Miedes et al., 2014; Shen et al., 2022).

In *C. chinense*, TSS accumulation appears to result from a broader metabolic transition that redirects carbon toward vacuolar reserves, independently of climacteric ethylene signaling. Among the genes identified here (Table 1), *CaSUS2, CaSWEET10*, *and CaAMY1/2* emerge as the strongest candidates for direct regulation of sugar transport, mobilization, and accumulation, rendering them priority targets for future functional validation. In contrast, the weak association between TSS and pigmentation‐related genes, coupled with the distinctive expression profile of the auxin‐conjugating gene *CaGH3.1*, suggests that sugar accumulation, carotenoid biosynthesis, and auxin homeostasis may be only partially coordinated processes. Interestingly, recent spatial transcriptomic studies have identified *AP2* and *PPR* transcription factors as important regulators of carotenoid biosynthesis in *Capsicum* (Reddy et al., 2025), indicating that additional upstream regulators linking fruit quality traits remain to be discovered. Clarifying how these regulatory networks integrate sugar accumulation, pigment biosynthesis, and hormone signaling will refine current models of fruit quality regulation in non‐climacteric Solanaceae crops, ultimately providing strategic targets for molecular breeding efforts aimed at improving fruit quality (Böttcher et al., 2011; Falagán & García‐Pastor, 2025; Peat et al., 2012; Sravankumar et al., 2018; Yuan et al., 2019).

## EXPERIMENTAL PROCEDURES

### Plant material and experimental conditions

To explore the metabolic and transcriptional determinants of TSS in *C. chinense*, we selected two contrasting accessions, A18 and A101, from the germplasm bank of the Federal University of Viçosa. These accessions originate from different geographic regions, A18 from Pindaré‐Mirim, Maranhão and A101 from Viçosa, Minas Gerais (Rosado‐Souza et al., 2015). In addition, two commercial genotypes were included as contrasting references: Habanero, a pungent cultivar of Mexican origin, and Biquinho, a non‐pungent Brazilian cultivar (Figure 1A). The accessions differ markedly in fruit pungency and color, providing a valuable contrast for investigating traits associated with fruit quality.

We conducted the experimental procedures as previously described (Rodrigues‐Salvador et al., 2022). Seeds were collected from field‐grown plants covered with white polyester fabric for at least two consecutive generations to ensure self‐pollination. Germination was performed on a commercial substrate (Tropstrato HT hortaliças, Vida Verde, Mogi Mirim‐SP, Brazil), and 25 days after sowing, seedlings were transplanted into 5 dm^3^ pots with a mixture of soil and substrate (1:1 w/w). Each pot was fertilized with 5.62 g of fertilizer 20‐5‐20 (N‐P_2_O_5_‐K_2_O, Heringer). Plants were grown in a greenhouse located in Viçosa, Minas Gerais, Brazil (642 m a.s.l., 20°45' S; 42°51' W), with regular irrigation and weekly fertilization of a solution containing 5 g L^−1^ of (NH_4_)_2_SO_4_ and 2.5 g L^−1^ of KCl, using 40 mL per pot.

### Fruit harvests

To determine the precise age of the fruits, we marked flowers at anthesis following the onset of flowering as previously described (Osorio et al., 2012). Pericarp samples from the four *C. chinense* accessions were collected at midday at two developmental stages: 20 and 60 days after anthesis (DAA). For each accession and stage, six fruits were collected for metabolic analysis and three fruits for RNA‐seq analysis. All samples were snap‐frozen in liquid nitrogen and stored at −80 °C until processing. Frozen samples were homogenized using a ball mill before metabolic profiling and transcriptomic analyses.

### Pigment quantification

We determined chlorophyll *a*, chlorophyll *b*, and total carotenoids as described by Wellburn (1994), with minor modifications. Approximately, 40 mg (fresh weight) pericarp powder was suspended in 2 mL of 80% acetone, incubated in darkness for 30 min, and centrifuged at 15000 ***g*** for 10 min at 4 °C. The absorbance of the supernatant was measured at 470, 646, and 663 nm using a spectrophotometer (VERSA max microplate reader).

### Metabolite profiling

We performed metabolite extraction, derivatization, internal standard addition, and sample injection for gas chromatography time‐of‐flight mass spectrometry (GC‐TOF‐MS: Agilent 7890 A GC system coupled to a Pegasus HT TOF/MS, LECO) according to Osorio et al. (2012). Chromatograms and mass spectra were processed using Chroma TOF 1.0 (LECO) and TAGFINDER 4.0 (Luedemann et al., 2008). Metabolite identification was performed using the Golm Metabolome Database (Kopka et al., 2005).

### Quantification of primary metabolites

The contents of starch, sucrose, fructose, and glucose in the pericarp tissues were determined following Fernie et al. (2001). Total protein and free amino acid levels were quantified as described (Cross et al., 2006). Malate and fumarate levels were determined as previously reported (Nunes‐Nesi et al., 2007). Total soluble solids (TSS) were quantified using a portable digital refractometer (ATC 106‐D Biobrix) expressed in °Brix (Lannes et al., 2007).

### Determination of phenolic compounds and secondary metabolites

Total soluble phenols were quantified using the Folin–Ciocalteu method (Sun et al., 2007). Individual secondary metabolites were quantified by high‐performance liquid chromatography (HPLC, Keinänen et al., 2001) with modifications. Ethanolic extracts were obtained from 50 mg of freeze‐dried pericarp tissue (Cross et al., 2006). After filtration on a 0.22 μm Millipore membrane, 50 μL aliquots were injected into an HPLC equipped with an Agilent ZORBAX Eclipse Plus C18 column (150 mm × 3.0 mm i.d., particle size 3 μm). Absorbance was recorded at 210, 254, 320, and 365 nm. The biphasic elution system consisted of phase A (0.25% of H_3_PO_4_ in ultrapure H_2_O, pH 2.2) and phase B (100% acetonitrile), with the following program settings: 0–6 min (0–12% B), 6–10 min (12–18% B), and 10–30 min (18–58% B), at a flow rate of 1 mL min^−1^. Identification and quantification of metabolites were performed using authentic standards and external calibration curves.

### RNA extraction

Total RNA was extracted from pericarp tissues at 20 and 60 DAA using TRIzol™ reagent (Invitrogen, Carlsbad, CA, USA; Cat. No. 15596018), following the manufacturer's instructions. Residual genomic DNA was removed by treatment with RQ1 RNAse‐Free DNase I (Promega, Madison, WI, USA, Cat. No. M6101), according to the manufacturer's instructions. RNA quality was assessed by agarose gel electrophoresis and spectrophotometry (Nano‐Drop, Wilmington, DE, USA). Total RNA integrity was evaluated using an Agilent 2100 Bioanalyzer (Agilent Technologies, Santa Clara, CA, USA) with the RNA 6000 Nano Kit (Cat. No. 5067–1511). Only samples with an RNA Integrity Number (RIN) higher than 8.0, an OD260/280 between 1.8 and 2.2 and an OD 260/230 ≥1.8 were selected for cDNA library construction. cDNA libraries were prepared using the TruSeq PE Cluster Kit v3‐cBot‐HS (Illumina, San Diego, CA, USA; Cat. No. PE‐401‐3001) according to the manufacturer's instructions. Sequencing was performed on an Illumina HiSeq™ 2500 using a 12‐h high‐output (HO) run to generate paired‐end 100 bp reads.

### RNA‐seq analysis

The RNA‐seq analysis pipeline is illustrated in Figure 1C. Briefly, raw sequencing reads were trimmed using fastp version 0.23.1 (Chen et al., 2018) to remove adapter sequences and low quality bases. Reads with a mean Phred quality score below 20 were filtered using a sliding window size of three. Quality control of pre‐ and post‐trimmed reads was also performed using fastp.

Trimmed reads were submitted to quasi‐mapping‐based quantification using Salmon (v1.6.0) (Patro et al., 2017) against a *C. annuum* Zunla‐1 (Qin et al., 2014) reference genome + transcriptome index. We used the *C. annuum* Zunla‐1 genome as reference due to the available *C. chinense* genome being flagged as contaminated, which raises concerns regarding its reliability for transcriptomic analyses. Also, the Zunla‐1 genome remains a well‐annotated and widely used reference in *Capsicum* studies (Han et al., 2023; Kim et al., 2021; Liu et al., 2023; Wang et al., 2020), supporting robust gene expression analyses and cross‐study comparability. Importantly, the validity of this approach was confirmed by ortholog‐based analyses between PI159236 (*C. chinense*) and Zunla‐1 (*C. annuum*) (Figures S4 and S5), which showed highly consistent expression patterns (Spearman's ρ > 0.84) across genotypes and developmental stages, indicating that the choice of reference genome did not bias the biological conclusions.

First, strict 1:1 orthologs were identified among the three accessions using OrthoFinder (v2.5.4) based on their annotated protein sequences. For each tissue‐stage combination (A18, A101, Biq, Hab at 20 and 60 DAA), mean TPM values per orthogroup were calculated by averaging across biological replicates. All subsequent analyses were performed on log_10 _(TPM + 1)‐transformed values to improve visualization and stabilize variance. Pairwise Spearman correlations between PI159236 and each of the other two accessions were computed for every condition, and the results were displayed as scatter plots with the diagonal *y* = *x* line for the comparison PI159236 x Zunla‐1. Additionally, DEGs in Zunla‐1 between stages (60 versus 20 DAA) were identified using DESeq2 (|log2FC| ≥1, *P*adj <0.05) and mapped to their corresponding orthogroups. In the scatter plots, DEGs were highlighted in blue, and separate Spearman correlations were calculated for the DEG subset. All figures are provided in the Supplementary Information. Salmon was run in paired‐end mode with automatic library type detection (‐l A). To improve quantification accuracy, sequence‐specific bias correction (‐‐seqBias) and GC‐content bias correction (‐‐gcBias) were enabled, and selective alignment was performed using the ‐‐validateMappings flag, which increases specificity by rescuing reads that would otherwise be discarded under strict quasi‐mapping. Mapping rates (Figure S3A) and library sizes (Figure S3B) were inspected for each sample via the log files generated during quantification and reported.

All 22 samples were processed independently, and output quantification files were subsequently imported into R version 4.2.0 (R Core Team, 2024) using the tximport package for downstream differential expression analysis. Raw read counts were filtered to remove genes with zero expression across all samples, summarized at the gene level, and DEGs were identified using DESeq2 version 1.36.0 (Love et al., 2014).

### Experimental design and statistical analysis

The experiment was conducted in a randomized block design, using five and three biological replicates per *C. chinense* accession for metabolite and RNA‐seq analyses, respectively. Each biological replicate consisted of tissue from at least three homogeneous fruits harvested from the same plant.

Metabolite data were analyzed by two‐way ANOVA (OLS) on log_2_‐transformed values, including genotype, stage (20 and 60 DAA), and their interaction. Estimated marginal means and Tukey's HSD pairwise comparisons (statsmodels) were used, with significance at adjusted *P* < 0.05; group differences were summarized by compact letter display (CLD). For heatmap visualization, z‐scores were calculated per metabolite from log_2_ emmeans by centering on the grand mean and scaling by the standard deviation across groups, enabling comparisons across metabolites with different units (GC–MS peak areas and molar concentrations). All heatmap analyses were conducted in Python using NumPy, SciPy, Pandas, and statsmodels.

In the RNA‐seq analysis, genes with FDR <0.05 (Benjamini & Hochberg, 1995) and |log_2_ fold change| ≥ 1 were considered differentially expressed. Metabolite–transcript Pearson correlation coefficients (PCCs) were calculated in R version 4.3.0 (R Core Team, 2024), and correlograms were generated for 20 and 60 DAA, retaining only significant correlations (FDR ≤0.05). Genes were grouped by functional classification, and correlograms were plotted using ggplot2 from the gplots package (Wickham, 2011).

## AUTHOR CONTRIBUTIONS

AN‐N conceived the project, designed research, and secured funding. AR‐S performed the experiments; AR‐S, RPO‐G, and L.R.‐S. performed biochemical analyses. WEBB analyzed the data and performed statistical analysis. AR‐S, WEBB, and RPO‐G wrote the first draft of the manuscript. FS, ARF, C dos SV, DGG, PMPV, DMR‐P, WLA, AZ, and AN‐N reviewed and contributed to the discussion of the manuscript. All authors read and approved the manuscript.

## CONFLICTS OF INTEREST

Regarding this study, the authors attest that there are no conflicts of interest.

## Supporting information


**Figure S1.** Genotype‐dependent regulatory amplitude over a shared ripening metabolic program drives divergent sugar accumulation, organic acid metabolism, and chromoplast differentiation across pungent and non‐pungent *C. chinense* accessions. Heatmap of Log_2_ FC of pigments, macromolecules, and metabolic profile in pericarp of fruits from four *C. chinense* accessions comparing 60 DAA against 20 DAA, in pungent (Hab and A101) and non‐pungent (Biq and A18) peppers. A color‐coded matrix represents the mean values of the Log_2_ FC across metabolites in five biological replicates of pericarp from pepper accessions. Blue and red squares represent negative and positive values, respectively. Pigments, starch, total proteins, total amino acids, and total soluble phenols were quantified spectrophotometrically. All other metabolites were quantified by GC–MS. Data were analyzed variable by variable using one‐way ANOVA followed by post‐hoc Tukey's HSD at 5% of significance.

**Figure S2.** Dynamics of soluble sugars and starch accumulation during *Capsicum* fruit development. Changes in the levels of (A) glucose, (B) fructose, (C) sucrose, and (D) starch across the assessed genotypes and developmental stages. Soluble carbohydrates (A–C) were profiled using GC–MS, and their abundances are expressed as the log2‐transformed relative response. Starch content (D) was quantified via an enzymatic assay and is presented as absolute concentration. Data represent the mean ± SE of five biological replicates. Statistical significance was determined by two‐way ANOVA followed by Tukey's post‐hoc test; different letters indicate statistically significant differences between groups (*P* < 0.05).

**Figure S3.** Comparative mapping rates of RNA‐seq reads against two distinct reference genomes. For each of the 22 *Capsicum* samples (genotypes A18, A101, Hab, and Biq at 20 and 60 days after anthesis), the percentage of reads that mapped to the corresponding reference genome is shown. (A) Data points indicate individual biological replicates with specific mapping percentages in black. Alignment to the PI159236 reference genome (orange) exhibits mapping rates between 47.7% and 58.0%. In contrast, alignment to the Zunla‐1 reference genome (red) shows consistently higher mapping rates, ranging from 72.7% to 85.6%. The absolute minimum and maximum mapping rates observed for each reference are highlighted in bold dark blue and red, respectively, while values on the far right represent the overall mean ± standard error (SE) across all samples. The overall high mapping efficiency against the Zunla‐1 genome confirms the good quality of the RNA‐seq data and supports the comparability of expression estimates across *Capsicum* genotypes. (B) The library sizes for each sample are indicated in the barplot.

**Figure S4.** Cross‐species transcriptome conservation between *C. chinense* (PI159236) and *C. annuum* (Zunla‐1) supports the validity of heterologous reference‐based quantification. Scatter plots display Spearman rank correlations of transcript abundance (TPM) for single‐copy orthogroups between PI159236 and Zunla‐1 at 20 DAA (A–D) and 60 DAA (E–H) across all four accessions (Hab, Biq, A18, and A101). Analysis was restricted to moderately to highly expressed genes (25th–75th TPM percentiles). Point colors indicate accession identity (red: Hab and Biq; orange: A18 and A101; blue: differentially expressed genes identified by DESeq2) and shapes denote developmental stage (triangles: 20 DAA; squares: 60 DAA). The dashed diagonal represents the *y* = *x* line. Spearman's ρ, *P*‐value, and number of orthogroups (*n*) are shown in each panel. High positive correlations across all accession‐stage combinations indicate strongly conserved ortholog expression patterns between species, validating the use of the Zunla‐1 reference transcriptome for quasi‐mapping‐based quantification of *C. chinense* reads.

**Figure S5.** Cross‐species transcriptome conservation between *C. chinense* (PI159236) and *C. annuum* (Zunla‐1) supports the validity of heterologous reference‐based quantification. Scatter plots display Spearman rank correlations of transcript abundance (TPM) for single‐copy orthogroups between PI159236 and Zunla‐1 at 20 DAA (A–D) and 60 DAA (E–H) across all four accessions (Hab, Biq, A18, and A101). Analysis was restricted to moderately to highly expressed genes (25th–75th TPM percentiles). Point colors indicate accession identity (red: Hab and Biq; orange: A18 and A101; blue: differentially expressed genes identified by DESeq2) and shapes denote developmental stage (triangles: 20 DAA; squares: 60 DAA). The dashed diagonal represents the *y* = *x* line. Spearman's ρ, *P*‐value, and number of orthogroups (*n*) are shown in each panel. High positive correlations across all accession‐stage combinations indicate strongly conserved ortholog expression patterns between species, validating the use of the Zunla‐1 reference transcriptome for quasi‐mapping‐based quantification of *C. chinense* reads.

**Table S1.** Metabolite abundance data and statistical analysis underlying the heatmap are shown in Figure 2. Five biological replicates were analyzed for each genotype‐stage combination (A18_20 DAA, A18_60 DAA, A101_20 DAA, A101_60 DAA, Biq_20 DAA, Biq_60 DAA, Hab_20 DAA, and Hab_60 DAA). Sixty‐three metabolites and metabolic features were quantified. For each compound, the table reports metabolite/feature abundance or concentration, *P*‐values from two‐way ANOVA + Tukey HSD, and log2 fold change values [log2(60 DAA/20 DAA)] calculated for each genotype. Abbreviation: DAA, days after anthesis.

**Table S2.** VST‐normalized transcriptome dataset across *Capsicum chinense* genotypes and developmental stages. Gene expression profiles were generated from pericarp tissue of four *C. chinense* genotypes (Biq, Hab, A101, and A18) sampled at 20 and 60 DAA. The dataset includes only genes that passed the filtering criteria of a minimum of 10 reads in at least two samples per experimental group. Most genotype‐stage combinations are represented by three biological replicates; A18_20 DAA and Hab_60 DAA are represented by two biological replicates. Gene annotation columns include GENE_SYMBOL, GENENAME, REFSEQ accession number, and ENTREZID identifier.

**Table S3.** Metabolite abundance and gene expression data used for the Pearson correlation analysis are shown in Figure 5A. This table contains the metabolite abundance and VST‐normalized gene expression values used to calculate the Pearson correlation coefficients for the 20 DAA correlogram. Data were obtained from four *C. chinense* genotypes (A18, A101, Biq, and Hab) and were used for the correlation analysis presented in Figure 5A.

**Table S4.** Metabolite abundance and gene expression data used for the Pearson correlation analysis are shown in Figure 5B. This table contains the metabolite abundance and VST‐normalized gene expression values used to calculate the Pearson correlation coefficients for the 60 DAA correlogram. Data were obtained from four *C. chinense* genotypes (A18, A101, Biq, and Hab) and were used for the correlation analysis presented in Figure 5B.

**Table S5.** Annotation and expression matrix of most variable genes across *C. chinense* accessions at 20 and 60 DAA. VST‐normalized expression values used for hierarchical clustering and heatmap construction in Figure 6, as well as the gene abbreviations used in Figure 6. The dataset encompasses expression profiles from individual biological replicates of four *Capsicum chinense* accessions (A18, A101, Hab, and Biq) sampled at 20 and 60 DAA, with replicate identities indicated by suffixes (_1, _2, and _3). The table includes gene identifiers (Gene Name, Gene Symbol, ENTREZ_ID, locus ID, and REFSEQ accession), assigned functional categories, and curated descriptions for the 51 fully annotated genes. These genes are associated with major biological processes underlying fruit development and ripening, including carbohydrate and lipid metabolism, cell wall remodeling, carotenoid biosynthesis, auxin and alkaloid metabolism, photosynthesis, cellular respiration, and defense responses. Positive and negative expression values reflect relative differences in transcript abundance, enabling direct visualization of stage‐ and genotype‐dependent transcriptional reprogramming.

## Data Availability

The data that support the findings of this study are available in the supplementary material of this article. The raw and processed sequence data reported in this paper have been deposited in the Gene Expression Omnibus (https://www.ncbi.nlm.nih.gov/geo/query/acc.cgi) under the accession number GSE326045.
