# Supplementary figures and images for: Integrated transcriptome–metabolome analyses reveal regulatory networks underlying soluble solids accumulation in Capsicum chinense fruits
Source: Plant J. 2026 Jul 9;127(1):e71020. doi: 10.1111/tpj.71020 (PMC13348266; doi:10.1111/tpj.71020)

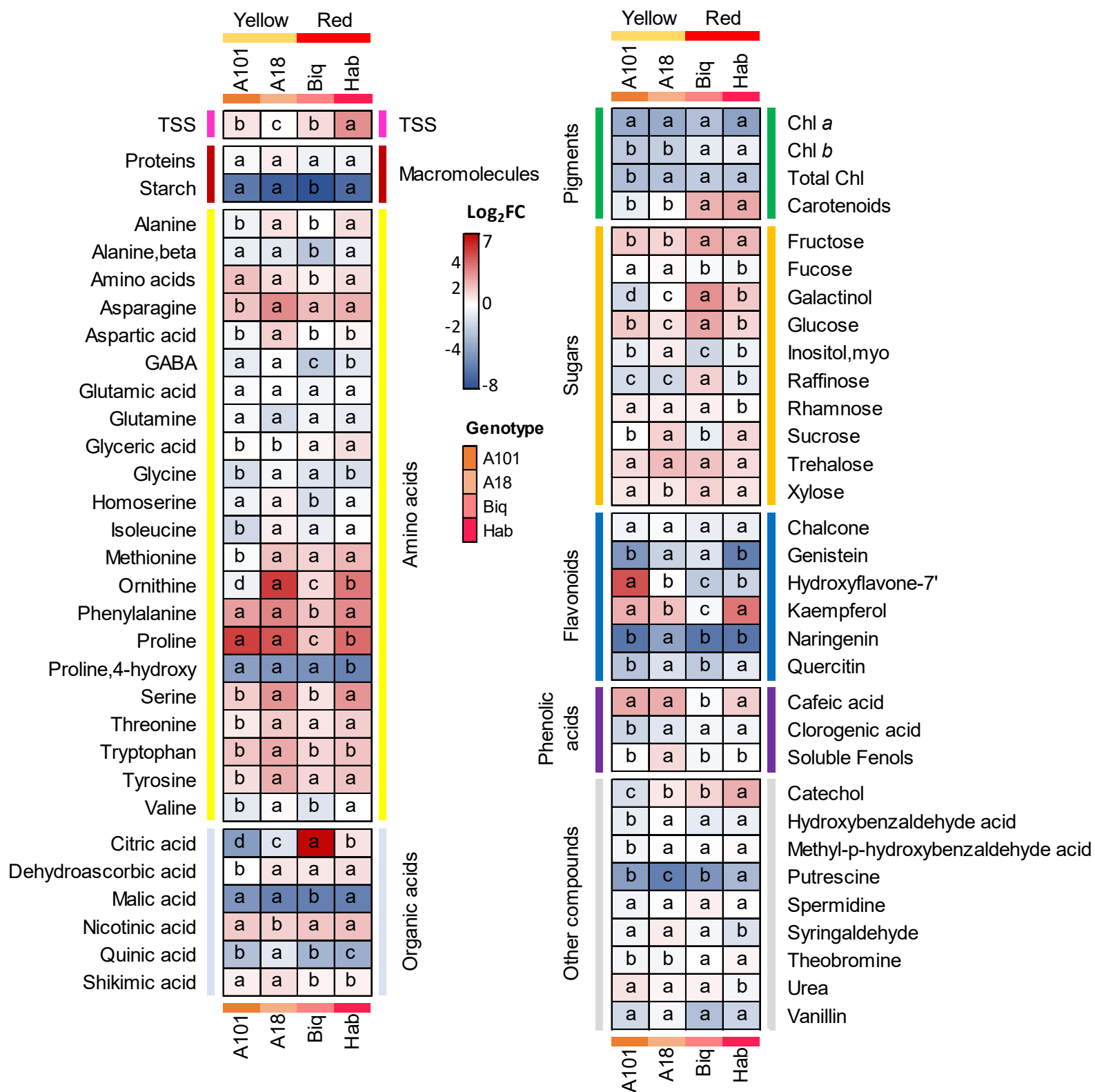

Supplement: Supplementary file 1 — Figure S1. Genotype‐dependent regulatory amplitude over a shared ripening metabolic program drives divergent sugar accumulation, organic acid metabolism, and chromoplast differentiation across pungent and non‐pungent C. chinense accessions. Heatmap of Log2 FC of pigments, macromolecules, and metabolic profile in pericarp of fruits from four C. chinense accessions comparing 60 DAA against 20 DAA, in pungent (Hab and A101) and non‐pungent (Biq and A18) peppers. A color‐coded matrix represents the mean values of the Log2 FC across metabolites in five biological replicates of pericarp from pepper accessions. Blue and red squares represent negative and positive values, respectively. Pigments, starch, total proteins, total amino acids, and total soluble phenols were quantified spectrophotometrically. All other metabolites were quantified by GC–MS. Data were analyzed variable by variable using one‐way ANOVA followed by post‐hoc Tukey's HSD at 5% of significance. [file TPJ-127-0-s007.pdf]

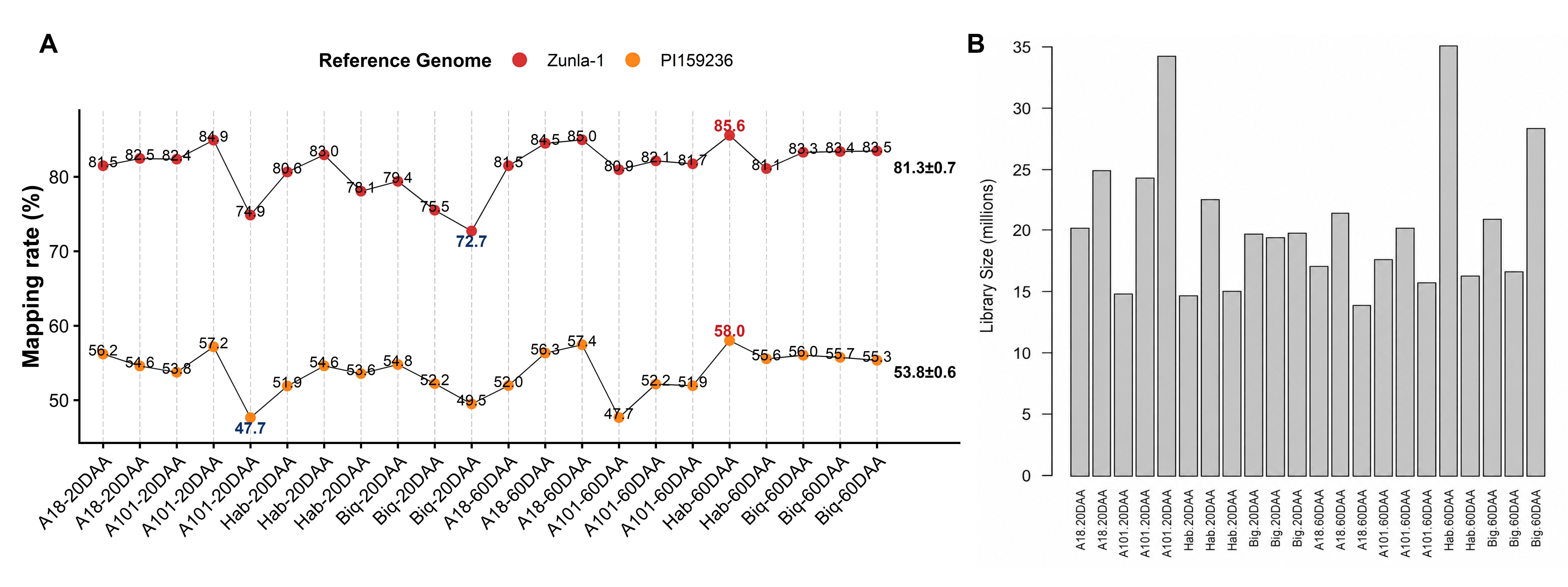

Supplement: Supplementary file 3 — Figure S3. Comparative mapping rates of RNA‐seq reads against two distinct reference genomes. For each of the 22 Capsicum samples (genotypes A18, A101, Hab, and Biq at 20 and 60 days after anthesis), the percentage of reads that mapped to the corresponding reference genome is shown. (A) Data points indicate individual biological replicates with specific mapping percentages in black. Alignment to the PI159236 reference genome (orange) exhibits mapping rates between 47.7% and 58.0%. In contrast, alignment to the Zunla‐1 reference genome (red) shows consistently higher mapping rates, ranging from 72.7% to 85.6%. The absolute minimum and maximum mapping rates observed for each reference are highlighted in bold dark blue and red, respectively, while values on the far right represent the overall mean ± standard error (SE) across all samples. The overall high mapping efficiency against the Zunla‐1 genome confirms the good quality of the RNA‐seq data and supports the comparability of expression estimates across Capsicum genotypes. (B) The library sizes for each sample are indicated in the barplot. [file TPJ-127-0-s008.png]

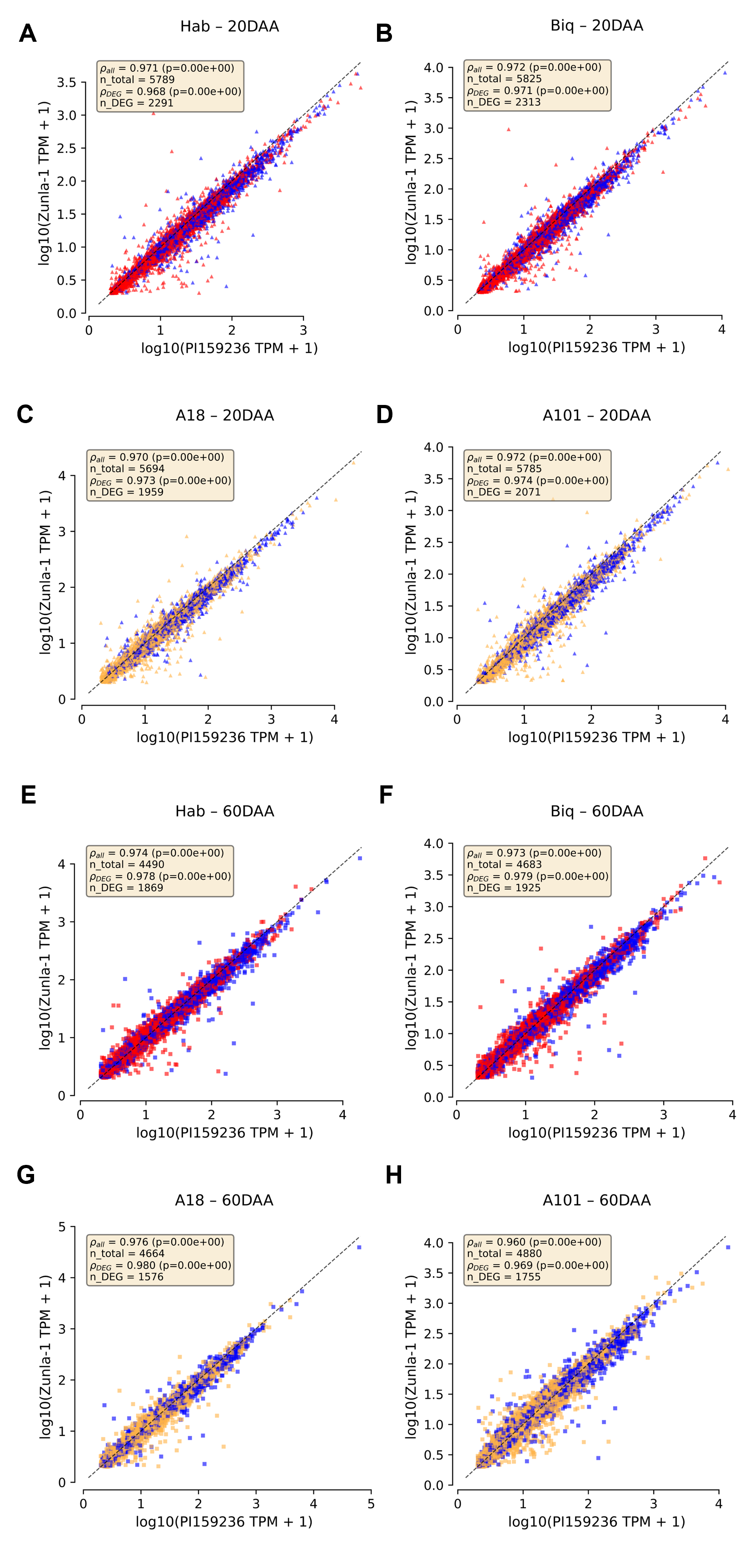

Supplement: Supplementary file 4 — Figure S4. Cross‐species transcriptome conservation between C. chinense (PI159236) and C. annuum (Zunla‐1) supports the validity of heterologous reference‐based quantification. Scatter plots display Spearman rank correlations of transcript abundance (TPM) for single‐copy orthogroups between PI159236 and Zunla‐1 at 20 DAA (A–D) and 60 DAA (E–H) across all four accessions (Hab, Biq, A18, and A101). Analysis was restricted to moderately to highly expressed genes (25th–75th TPM percentiles). Point colors indicate accession identity (red: Hab and Biq; orange: A18 and A101; blue: differentially expressed genes identified by DESeq2) and shapes denote developmental stage (triangles: 20 DAA; squares: 60 DAA). The dashed diagonal represents the y = x line. Spearman's ρ, P‐value, and number of orthogroups (n) are shown in each panel. High positive correlations across all accession‐stage combinations indicate strongly conserved ortholog expression patterns between species, validating the use of the Zunla‐1 reference transcriptome for quasi‐mapping‐based quantification of C. chinense reads. [file TPJ-127-0-s001.tif]

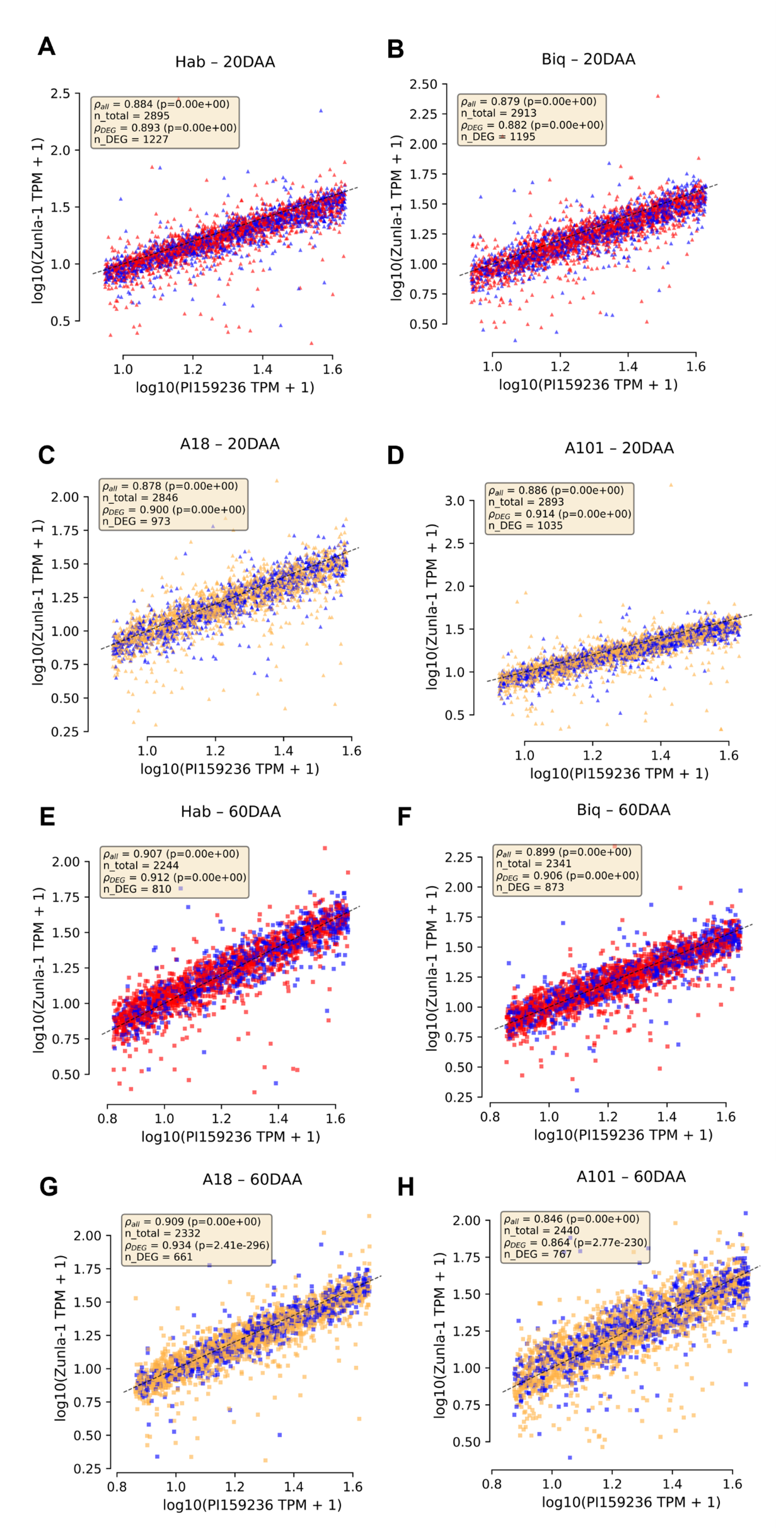

Supplement: Supplementary file 5 — Figure S5. Cross‐species transcriptome conservation between C. chinense (PI159236) and C. annuum (Zunla‐1) supports the validity of heterologous reference‐based quantification. Scatter plots display Spearman rank correlations of transcript abundance (TPM) for single‐copy orthogroups between PI159236 and Zunla‐1 at 20 DAA (A–D) and 60 DAA (E–H) across all four accessions (Hab, Biq, A18, and A101). Analysis was restricted to moderately to highly expressed genes (25th–75th TPM percentiles). Point colors indicate accession identity (red: Hab and Biq; orange: A18 and A101; blue: differentially expressed genes identified by DESeq2) and shapes denote developmental stage (triangles: 20 DAA; squares: 60 DAA). The dashed diagonal represents the y = x line. Spearman's ρ, P‐value, and number of orthogroups (n) are shown in each panel. High positive correlations across all accession‐stage combinations indicate strongly conserved ortholog expression patterns between species, validating the use of the Zunla‐1 reference transcriptome for quasi‐mapping‐based quantification of C. chinense reads. [file TPJ-127-0-s010.tif]
